# Continuous Twin Screw Granulation: A Review of Recent Progress and Opportunities in Formulation and Equipment Design

**DOI:** 10.3390/pharmaceutics13050668

**Published:** 2021-05-07

**Authors:** Christoph Portier, Chris Vervaet, Valérie Vanhoorne

**Affiliations:** Laboratory of Pharmaceutical Technology, Department of Pharmaceutics, Ghent University, Ottergemsesteenweg 460, B-9000 Ghent, Belgium; Christoph.Portier@UGent.be (C.P.); Chris.Vervaet@UGent.be (C.V.)

**Keywords:** continuous manufacturing, twin screw granulation, wet granulation, formulation development, process development, innovation, review

## Abstract

Continuous twin screw wet granulation is one of the key continuous manufacturing technologies that have gained significant interest in the pharmaceutical industry as well as in academia over the last ten years. Given its considerable advantages compared to wet granulation techniques operated in batch mode such as high shear granulation and fluid bed granulation, several equipment manufacturers have designed their own manufacturing setup. This has led to a steep increase in the research output in this field. However, most studies still focused on a single (often placebo) formulation, hence making it difficult to assess the general validity of the obtained results. Therefore, current review provides an overview of recent progress in the field of continuous twin screw wet granulation, with special focus on the importance of the formulation aspect and raw material properties. It gives practical guidance for novel and more experienced users of this technique and highlights some of the unmet needs that require further research.

## 1. Introduction

**Figure 1:**
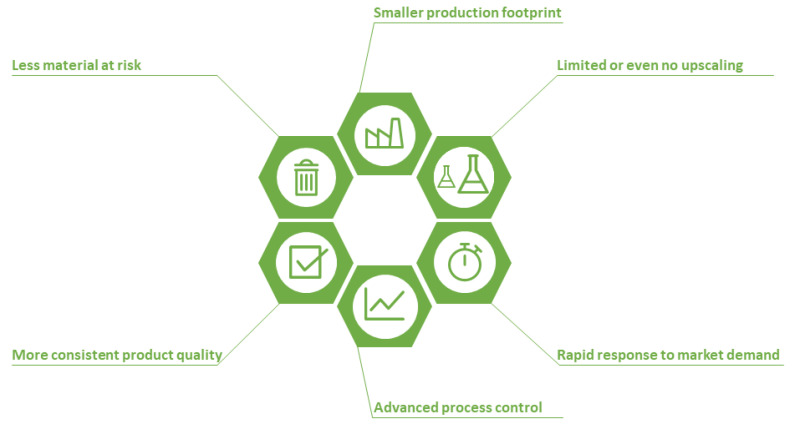
Advantages of continuous manufacturing.

Continuous manufacturing techniques have rapidly gained interest in the pharmaceutical industry in the last ten years, due to their widely recognized advantages (Figure 1). Most importantly, continuous manufacturing techniques often eliminate the necessity of scaling up between initial clinical manufacturing and the final commercial production, because development is already performed on commercial scale equipment [1,2,3]. As the amount of material processed in continuous manufacturing can easily be adapted by changing the duration of production, this accounts for the rapid response to changing market demand [4,5]. This is in clear contrast to conventional batch manufacturing where batch size is purely restricted by the dimensions of the equipment [6].

In a continuous manufacturing line, advanced process control is achieved by implementing process analytical technology (PAT) and soft sensors throughout the system, consequently enabling real time release strategies and a shorter time to market. Non-conforming material can be tracked and eliminated from the continuous manufacturing line at designated diversion points [4,7,8,9,10]. For more information on PAT implementation, advanced control strategies, and regulatory viewpoints, readers are referred to the recent review of Vanhoorne and Vervaet [11]. In addition to a more consistent drug product quality, only a limited amount of material is at risk, as only a fraction of the material is present in a single unit operation within the manufacturing line. In batch manufacturing techniques, significantly larger amounts of material are present in each unit operation (e.g., blending, high shear granulation), putting the entire batch at risk of rejection [12]. Additional economic advantages have been attributed to continuous manufacturing due to a smaller production footprint and elimination of intermediate product storage [13,14].

Given the complexity of most continuous manufacturing lines, enhanced process knowledge and quality-by-design (QbD) are of high interest towards drug product development via a science and risk-based approach. Using design of experiments (DOE), the effect of several factors can be evaluated simultaneously, hereby identifying critical process parameters (CPP) and critical material attributes (CMA), subsequently generating predictive models. Based on these models and knowledge on process stability, a design space can be defined where the quality target product profile (QTPP) is met [15,16]. Hence, the quality of the product is built into the process rather than being tested at the end of the manufacturing process. Given the continuous nature and short residence time of the unit operations such as twin screw granulation, changes in raw material properties and process settings easily propagate throughout the system and are quickly detected, hence allowing fast data and knowledge generation. This is one of the main reasons why QbD approaches are especially suited for continuous manufacturing processes [17].

Integrated continuous manufacturing lines for tablets are generally designed to handle direct compression, wet granulation, and dry granulation manufacturing pathways [3,18]. Direct compression is considered the easiest and cheapest pathway as no intermediate granulation steps are involved. Furthermore, raw materials are not subjected to moisture or heat, which reduces the risk of degradation. However, this pathway requires the blend to have favorable flowability and compaction properties as well as high content uniformity [18,19,20,21]. To overcome these requirements, intermediate granulation steps (mostly wet and dry granulation) can be implemented.

Granulation is a commonly applied particle enlargement technique that improves detrimental raw material properties (powder flow, density, cohesiveness, electrostatic charging) and ensures a consistent and homogeneous API distribution [20,22]. Based on the European Public Assessment Reports (EPAR), Leane et al. found that >70% of tablet formulations with a specified manufacturing pathway include a wet (55%) or dry (16%) granulation step [20]. Dry granulation techniques such as roller compaction are often preferred over wet granulation, as these eliminate an additional drying step and are more suitable for moisture-sensitive APIs [23,24]. Wet granulation techniques such as twin screw wet granulation are mainly implemented to obtain a more uniform distribution of the formulation ingredients and when compaction properties do not allow granulation through roller compaction [17]. Fülop et al. recently demonstrated the superior API and liquid distribution behavior in twin screw wet granulation compared to high shear batch granulation for a very low-dosed carvedilol formulation [25].

Compared to batchwise wet granulation techniques such as high shear granulation, twin screw granulation requires significantly less water to obtain granules of a desired size [26,27]. Generally, less spherical and more porous granules are produced than via high shear granulation. These granule properties are favorable during granulation as more fragmentation occurs, hence increasing tablet tensile strength [28,29,30]. Granules prepared via twin screw wet granulation often have intermediate granular strength and density, compared to high shear and fluid bed granulation [25,30].

**Figure 2:**
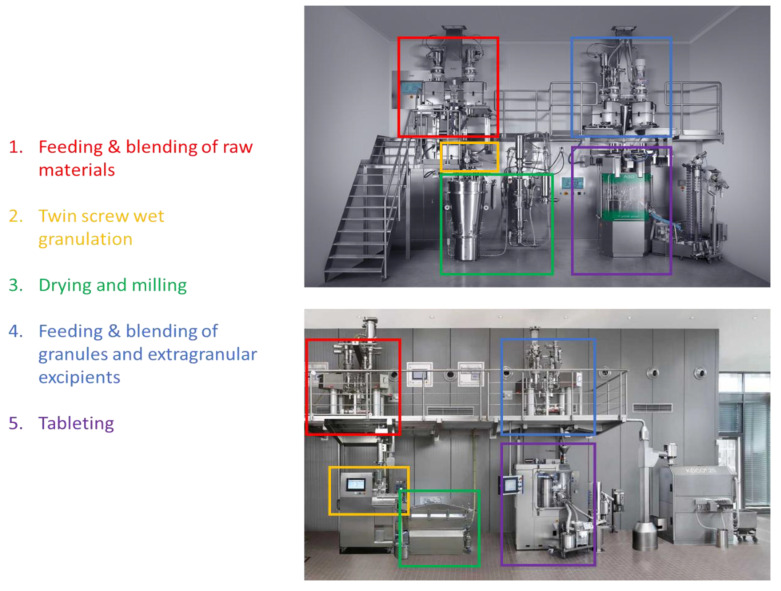
Setup of two fully integrated powder-to-tablet lines. (**Top**): Consigma by GEA Pharma Systems (adopted from www.gea.com; accessed on 9 April 2021); (**bottom**): QbCon by L.B. Bohle (courtesy of L.B. Bohle).

In a fully integrated powder-to-tablet twin screw wet granulation line (Figure 2), raw materials (excipients and APIs) are fed individually to a continuous inline blender using a series of gravimetric feeders. The resulting blend is fed into a twin screw granulator where wet granules are formed. These granules are gravimetrically or pneumatically transferred into a (semi-) continuous drying unit and subsequently milled to obtain the desired particle size distribution. In a next step, these dried and milled granules are blended with extragranular excipients such as magnesium stearate, added using gravimetric feeders. This finalized blend is further processed using a rotary tablet press. Subsequently, tablets are dedusted and potentially coated before being transferred to a packaging line.

Despite the fact that some of these techniques have already been implemented for several decades in other industries such as the (petro)chemical and food manufacturing [3,31,32], pharmaceutical implementation of these techniques was initially hindered by the inappropriate scale of the available equipment as well as the lack of a clear regulatory framework. The development of fully integrated manufacturing lines (Figure 2) by equipment manufacturers such as GEA Pharma Systems, L.B. Bohle, and Glatt was a key driver towards adoption and implementation by global pharmaceutical companies.

Following GEA Pharma Systems, which has been one of the pioneers for the launch of pharmaceutical continuous manufacturing equipment, several equipment vendors ventured into the field of continuous manufacturing. L.B. Bohle made significant investments towards the development of its QbCon line, which has a similar setup compared to that of the GEA Consigma-25 line. With a multi-million investment in 2014 to set up the Bohle Technology Center, it was clear that the focus of this equipment vendor shifted from batch to continuous manufacturing [33]. In recent years, L.B. Bohle has further developed its portfolio with the launch of the mobile QbCon 1, which initially had a similar setup to the GEA Consigma-1 but was later refined by replacing the semi-continuous fluid bed dryer with a fully continuous fluid bed drying system. Similar investments have been made by other equipment vendors such as Glatt, Fette, and Syntegon. Whereas Glatt and Fette further ventured in the field of conventional continuous manufacturing, Syntegon has chosen to adopt an approach focusing on the production of mini-batches using a series of small parallel fluid bed granulators and dryers. Their platform, Xelum, combines some of the well-established advantages of continuous manufacturing, such as lack of scale-up with batch advantages such as avoiding transfer of wet granules, increased dosing efficiency, and full traceability [34].

In recent years, major global pharmaceutical companies such as Janssen, Vertex, Lilly, and Pfizer have gained market approval for several drug products produced through direct compression and twin screw wet granulation [35,36,37,38,39]. Since the initial publication of the U.S. Food and Drug Administration (FDA) Guidance for Industry on PAT in 2004 [40], significant steps have been taken by regulators such as FDA, EMA, and ICH to facilitate the use of novel manufacturing techniques. With the publication of the FDA draft guidance for industry on quality considerations for continuous manufacturing [1], it became clear that the importance of implementing continuous manufacturing techniques is recognized on a global scale.

In addition to global pharmaceutical companies and equipment vendors, leading excipient manufacturers such as BASF, DFE, DOW, JRS, and Roquette have recently also shown their interest to venture into the field of continuous manufacturing [41,42,43,44,45,46]. Due to the inherent technological differences compared to batch manufacturing, novel material grades could prove beneficial for several unit operations within a continuous manufacturing line such as feeding, blending, granulation, and tableting. Since residence time in most of these unit operations is limited compared to batch manufacturing, a fast and constant performance of all raw materials is required [47,48,49].

**Figure 3:**
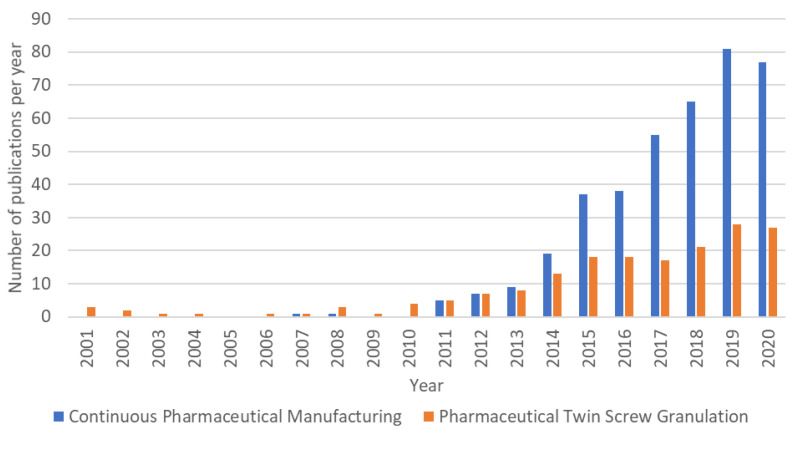
Evolution in publications on continuous pharmaceutical manufacturing and pharmaceutical twin screw granulation, indexed in PubMed as Continuous-Manufacturing AND Pharmaceutical (blue) and Pharmaceutical AND Twin-Screw AND Granulation (orange), respectively.

The increased interest of the industry in continuous manufacturing has also led to several partnerships with academic institutes and consortia such as CESPE, C-SOPS, CMAC, and the European Consortium for Continuous Pharmaceutical Manufacturing. These partnerships have been one of the major drivers accounting for the increased research output in this field in the last ten years as shown in Figure 3.

Considering continuous twin screw granulation, initial studies mainly focused on the effect of process settings [49,50,51] and screw configurations [52,53,54]. However, more recent studies have highlighted the importance of formulation and raw material properties [2,12,27,46,47,55,56,57,58,59,60,61] and aspects related to drug product registration such as PAT implementation and control strategies [7,10,62]. Since most of the studies on twin screw wet granulation focus on a single formulation, often not containing an active pharmaceutical ingredient (API), results are difficult to generalize. Therefore, the current review gives an overview of recent advances in the field of continuous twin screw wet granulation, with special consideration for the importance of formulation aspects and raw material properties in drug-loaded formulations.

## 2. Influence of Raw Material Properties

### 2.1. Fillers

#### 2.1.1. Commonly Used Fillers and Filler Combinations

Compared to batch manufacturing, similar excipients are often used in twin screw wet granulation, both in academic research as in industry. In commercial formulations, containing one or several APIs, often a combination of a water soluble filler and microcrystalline cellulose (MCC) is included. This filler combination has been extensively studied in literature, with lactose being the most used water soluble filler (Table 1). The water soluble filler will partially dissolve inside the granulator barrel and form solid bridges between the powder particles during the subsequent drying phase [57,63]. MCC is added as this contributes to the robustness of the formulation, making it less susceptible towards deviations in process settings and altered API properties [12,57]. Additionally, MCC has a clear beneficial effect during tableting as it deforms plastically, maximizing the area of interparticulate bonding and increasing tablet tensile strength [64,65]. Generally, the ratio of the water soluble filler to MCC is above 1 to avoid excessive water addition required by MCC. Due to the continuous nature of the subsequent drying process, residence time and drying capacity are limited, restricting the amount of water that can be evaporated [59].

Because of the high water binding capacity and batch-to-batch variability of MCC [50,71], lactose has also been studied as the single filler in drug-loaded formulations [14,15,25,57,72,73]. In contrast, Schmidt et al. reported granulation of ibuprofen without a water soluble filler, incorporating MCC as the single filler. As the API was added as a suspension, the high water binding capacity of MCC was utilized to allow the use of high liquid-to-solid (L/S) ratios (0.5–0.9), hence obtaining a higher drug load [74].

#### 2.1.2. Microcrystalline Cellulose

MCC is a commonly used excipient in both batch as continuous wet granulation techniques, which is produced through partial hydrolysis of cellulose [50,63]. However, significant batch-to-batch variability has been observed due to its natural origin. Fonteyne et al. found that MCC batches had varying water binding capacity, which originated from a different degree of crystallinity. As binder addition could not mitigate these differences, it was concluded that differences in MCC batches should be taken into account during manufacturing [50]. Similar differences in water binding capacity were observed by Portier et al. Based on extensive raw material characterization, they adopted a multivariate approach to assess the effect of MCC raw material attributes during granulation and subsequent fluid bed drying. It was shown that MCC batches with a low water binding capacity, low moisture content, and high bulk density are preferred for twin screw wet granulation. In addition, a quantitative approach towards mitigating batch-to-batch variability of raw materials was proposed [71].

#### 2.1.3. Lactose

Like MCC, lactose is one of the most widely used excipients in twin screw wet granulation. El Hagrasy et al. demonstrated that limited differences in particle size distribution (PSD) were obtained when comparing three distinct lactose grades (Pharmatose 200M, Supertab 30GR and Lactose Impalpable). However, the porosity of the formulation containing granular lactose (Supertab 30GR) was significantly lower at higher L/S ratios [75]. A multivariate approach describing the different granulation behavior of multiple lactose grades (Granulac 200, Granulac 70, Prismalac 40, and Flowlac 90) was reported by Hwang et al. Granule properties were dependent on the lactose grade. Granule friability of Granulac 200 was lower than friability of the other lactose grades at a low L/S ratio due to its large surface area and higher interaction between the granulation liquid and powder. However, at higher L/S ratios this effect was nullified [76].

#### 2.1.4. Mannitol

Mannitol is a polyol with an aqueous solubility similar to lactose [63]. It is sometimes used as a water soluble excipient instead of lactose (Table 2) due to its higher dissolution rate [47]. Mannitol is chemically more inert compared to lactose, as mannitol is not prone to Maillard type condensation reactions with primary and secondary amine functional groups in APIs [57,77]. Additionally, mannitol is well-known for existing in several polymorphic forms (α, β, and δ). Vanhoorne et al. reported a polymorphic transition from δ- to β-mannitol (Parteck Delta M) during twin screw granulation, resulting in enhanced plastic deformability and superior tabletability of the resulting granules [47]. This was later confirmed with a formulation containing 75% acetaminophen. Although no binders could be used as these inhibited the polymorphic transition, strong granules were obtained, showing only limited breakage and attrition during drying. [60]. A different polymorphic transition from α- to β- mannitol was described for a different grade of mannitol (Pearlitol 200SD), which was again accompanied by an altered morphology and higher specific surface area, accounting for the better tabletability [27].

### 2.2. Binders

#### 2.2.1. Immediate Release

The most commonly used immediate release binders in twin screw wet granulation of drug-loaded formulations are cellulose derivatives such as hydroxypropylmethylcellulose (HPMC) [12,21,57,66] and hydroxypropylcellulose (HPC) [26,29,67,68,78,79] as well as synthetic polymers such as polyvinylpyrrolidone (PVP) [14,25,59]. Although cellulosic binders are also commonly used in batch manufacturing, they are not always the most suitable choice for twin screw granulation. As the average residence time within the granulator barrel is limited to 5–20 s, it is important that binders are activated and obtain their full binding potential in a short timeframe. This contrasts with batch processes, where granules are formed over a time period of (tens of) minutes, and subsequently there is less need for fast wetting kinetics and binder activation. Portier et al. demonstrated that changing the binder from HPMC (Methocel E15 LV) to PVP (Kollidon K30) in twin screw wet granulation yielded significantly stronger granules at similar to lower L/S ratios when granulating a high-dosed poorly soluble, poorly wettable API [59]. The same PVP grade has also been reported for granulating formulations containing theophylline [14] and carvedilol [25]. Similar to the findings of Portier et al., Ritala et al. previously described a beneficial effect of PVP over HPMC to reduce the required L/S ratio for the granulation of dicalcium phosphate in high shear wet granulation [80]. Despite the potential benefit of PVP, formulators should take into account the presence of reactive impurities such as peroxide residues originating from the chemical synthesis. Consequently, this could induce degradation of APIs that are sensitive to oxidation [63,81].

Alongside these commonly used binders, Vandevivere et al. demonstrated the applicability of native starches as in situ binders when used in combination with dicalcium phosphate [45]. In a follow-up study focusing on the same filler, binder properties of several frequently and less frequently used binders were linked to granule friability. Good wettability of the formulation by the binder proved essential in addition to a high binder viscosity and low surface tension [46] As dicalcium phosphate is a poorly water soluble filler, it does not contribute to bond formation within the granules. Subsequently, all the observed granular binding effect could be attributed to the studied binders. For highly soluble formulations, Vandevivere et al. recommended the use of binders with low viscosity, fast dissolution kinetics, low surface tension, and good wetting of the formulation by the binder [58].

Apart from choosing an appropriate binder, the binder addition method can also have an impact on granule properties. El Hagrasy et al. described a significant reduction of the amount of fines by adding the binder (HPMC) as a wet binder dispersion instead of a dry powder ingredient in the premix [75]. Other authors found similar effects for PVP [51,82], HPMC [59,82], and HPC [82]. However, no consensus in literature exists on the effect of the binder addition method, as Vandevivere et al. reported a lower granule friability when applying dry binder addition of HPMC or PVP for granulating dicalcium phosphate [46]. Despite the potential gain in granule quality, formulators should be aware that the amount of binder that can be added is sometimes restricted by the viscosity of the resulting binder dispersion. In addition, the manufacturing process will also become more complex, and a higher amount of material could be at risk if failure occurs due to poor binder dispersion.

#### 2.2.2. Sustained Release

Currently, literature on sustained release using twin screw wet granulation is still limited to a few publications. Two studies were published by Vanhoorne et al. describing the use of several HPMC grades as matrix formers for sustained release. In a first study, 20% metoprolol tartrate was granulated using 20% HPMC 90SH-4000. Release of the API could be sustained over 16 h and did not depend on formulation and process settings. Whereas lactose or lactose and native maize starch as filler yielded granules with a regular shape, MCC as filler resulted in elongated granules at high L/S ratios. It was therefore concluded that the combination of HPMC and MCC is not preferred for sustained release [83]. Significantly more elongated granules were previously described by Thompson and O’Donnell who studied Methocel K4M and Kollidon SR as controlled release agents in a 5–20% concentration range [56]. The different granule elongation between both studies could be attributed to the different clearance between the granulator screws and barrel as different equipment types were used. Additionally, Thompson and O’Donnell used foam delivery of the granulation liquid (HPMC E3PLV), whereas Vanhoorne et al. added distilled water as granulation liquid using peristaltic pumps and nozzles.

A second study by Vanhoorne et al. compared the performance of several HPMC grades (Metolose 90SH-4000-SR, 90SH-100000-SR and 60SH-4000) as sustained release excipients in a concentration range of 20–40%. Sustained release of the API (20% theophylline) could be maintained over 24 h by changing the viscosity and substitution degree of the HPMC grade. It was, however, observed that the API was not homogeneously distributed across all size fractions [73]. This inhomogeneous distribution could originate from the swelling of HPMC in combination with the limited residence time within the barrel, resulting in insufficient mixing and subsequently varying API content across granule size fractions. Similarly, an inhomogeneous theophylline distribution was also described by Fonteyne et al. for an immediate release formulation where significantly more API was found in the coarser granule fractions [14].

### 2.3. Surfactants

Limited information on the use of surfactants in twin screw wet granulation is currently available. Portier et al. demonstrated that the addition of 0.2% sodium lauryl sulphate (SLS) to a formulation containing 50% of a poorly soluble API (mebendazole) had a clear beneficial effect on the required L/S ratio for granulation. A 20% reduction of the L/S ratio was achieved without affecting the physical properties of the granules (PSD, density and friability) [59]. This effect could be beneficial for formulations where the required L/S ratio is a limiting factor for the achievable throughput due to the limited drying capacity of the subsequent drying unit. A higher concentration of SLS (1.9%) was reported by Roggo et al., who evaluated a formulation containing 40% API [84].

Similarly, Schmidt et al. described the effects of polysorbate 80 addition on the required L/S ratio. It was observed that higher amounts of polysorbate 80 increased the solubility of the API (ibuprofen), which lowered the onset of paste formation. However, in this study, no data were provided about the effect on physical granule properties [74]. In contrast, Dhenge et al. found that higher concentrations of SLS did not reduce the required amount of water for granulation. It was observed that changing the amount of SLS (and therefore surface tension of granulation liquids) had no significant influence on the PSD, flow properties and granule strength [85].

In conclusion, the beneficial effect of surfactants on the required amount of liquid is formulation- and surfactant-dependent, and additional research is required. When implementing surfactants, the impact on dissolution of the drug product and bioavailability of the API should also be taken into account [18,86,87,88], as limited information on this topic is currently available within the field of twin screw wet granulation.

### 2.4. APIs

In this section, an overview is provided on the effect of API properties on granulation behavior, evaluating studies on API material variability as well as on the addition of APIs with diverse properties. Fonteyne et al. evaluated the impact of raw material attributes of seven grades of theophylline anhydrous (30%) on granule properties. They observed that coarser API particles resulted in larger granules and a reduced fraction of fines. Despite premixes with these coarser grades having significantly higher bulk and tapped density than their finer counterparts, differences in granule bulk and tapped density were much smaller. Surprisingly, granules produced with finer theophylline grades, which had the lowest raw material bulk and tapped density, had the highest bulk and tapped granule density. Granule shape, true density, and flowability were not significantly affected by raw material properties [14].

Stauffer et al. analyzed the impact of batch-to-batch variability of eight API batches produced using different synthetic routes and downstream processing. An integrated DOE approach was used, integrating raw material variability through the principal component scores, obtained in principal component analysis (PCA). A clear relation between the first two principal components (PC) and granule quality attributes was found. The first principal component was mainly driven by API crystal length, agglomerate size, flowability, and electrostatic charging. PC2 accounted for the variability between API batches due the span of the particle size distribution and the strength of the agglomerate. The third PC, dominated by surface energy, had no effect on granule quality attributes [78]. In a follow-up study, it was demonstrated that the API batch-to-batch variability could be mitigated by choosing an appropriate L/S ratio [79].

**Figure 4:**
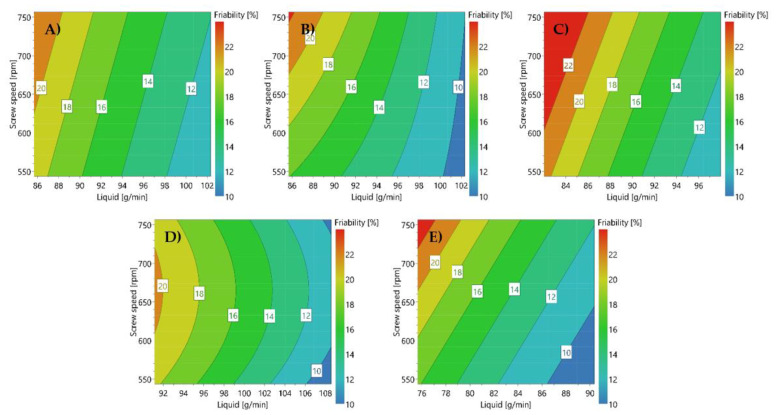
Friability contour plots of formulations containing 5% HPMC, lactose/MCC (1:1) as filler and (**A**) 5% acetaminophen, (**B**) placebo, (**C**) 5% theophylline anhydrous, (**D**) 10% mebendazole, and (**E**) 10% metformin hydrochloride. Adapted with permission from [12], Elsevier, 2020.

Granulation behavior of formulations containing 25% allopurinol, metformin.HCl, or acetaminophen was compared by Kyttä et al. They found that the APIs, varying significantly in aqueous solubility and particle size, affected the optimal water amount in twin screw wet granulation. However, a similar response behavior of the formulations containing these model APIs was observed [29]. Similar results were obtained by Portier et al., who described the robustness of a lactose/MCC filler in a 1:1 ratio combined with HPMC as binder towards handling APIs with diverse properties. In this study, the behavior of 5–10% API (mebendazole, metformin.HCl, acetaminophen, or theophylline) was compared to that of a placebo formulation. It was concluded that by applying a suitable L/S ratio, a similar response behavior was obtained, as illustrated in Figure 4. For APIs with similar granulation properties (theophylline and acetaminophen), almost identical response behavior was observed, indicating the potential use of surrogate APIs in formulation and process development [12].

Li et al. studied the impact of API properties in a twin screw granulation setup utilizing foamed binder delivery in formulations with a 15% drug load. The formulations containing the more hydrophobic APIs (griseofulvin or ibuprofen) required higher L/S ratios to achieve granules comparable to formulations comprising more hydrophilic APIs (acetaminophen or caffeine). However, a uniform API distribution was obtained, independent of the API properties, which was hypothesized to originate from the foamed binder addition method [66]. However, since no comparative data were provided using conventional wet binder addition on the same formulations, confirmation of this hypothesis is still required. The foamed binder addition method was previously introduced by Thompson et al. [89] and has subsequently been studied in depth by this research group [54,56,90,91,92,93].

## 3. Influence of Process Settings

Although the influence of process settings is not the main focus of this review, a basic overview is provided hereafter to give the reader a comprehensible insight in the major influential process settings in twin screw wet granulation and their interplay with formulation characteristics. For more detailed information on the impact of process settings on granule characteristics, the reader is referred to reviews of Seem et al. and Thompson et al. [32,94].

### 3.1. L/S Ratio

L/S ratio has been extensively described as the most influential factor in achieving granules with desired quality attributes. Increasing the L/S ratio reduces the amount of fines, increases bulk and tapped density, and generates particles with a higher strength and superior flow properties [2,32,50,57,75,95]. Water soluble excipients are more susceptible to changes in the L/S ratio compared to insoluble excipients such as MCC [57,76,96]. In general, L/S ratio can be considered the most critical process setting, which is perfectly suited as a screening tool to assess the manufacturability of a given formulation via twin screw wet granulation. Given the short residence time within the granulator, an immediate response in granule quality is observed when changing the L/S ratio.

### 3.2. Screw Speed

Screw speed is one of the main influential factors regarding the barrel fill level. The effect of screw speed on particle characteristics is inconsistent in literature, possibly indicating a formulation-dependent behavior. Additionally, differences in extruder types (e.g., different clearance between screws and barrel) and evaluated screw speed ranges could account for the contradicting research findings [32]. Dhenge et al. found that granule size slightly increased at lower screw speeds due to the longer residence time, allowing more granule growth [97]. Portier et al. also reported a beneficial effect of operating at a lower screw speed when considering granule flowability, strength, and the fraction of fines of four low-dosed formulations. However, for a high drug load mebendazole formulation, higher screw speeds proved favorable, similar to the observations of Thompson and O’Donnell [56]. It was hypothesized that some formulations benefit from the increased densification at lower screw speed, whereas other formulations are more susceptible to the higher kinetic energy that is transferred into the system at high screw speed [57]. Several other studies evaluating the impact of screw speed reported a negligible effect on granule properties [51,83,98,99]. Overall, it is concluded that screw speed can often be used to steer granule quality. However, a sufficiently high screw speed should always be applied to avoid excessive barrel fill, resulting in clogging of the powder inlet and high torque. Especially for formulations benefiting from a high barrel fill level and hence low screw speed, these contradicting preferences should be sufficiently balanced.

### 3.3. Throughput

In addition to screw speed, material throughput also determines the barrel fill level. However, reported effects on granule characteristics are often less pronounced compared to the impact of screw speed. Several authors observed an increase in granular density, size, and/or strength at higher throughput, originating from the higher compressive forces generated in the kneading blocks [15,48,94,97,100,101]. In contrast, other studies reported no relevant effect of material throughput on granule attributes [2,51,57] or even a reduction of granule size [83]. This is again an indication of formulation-, equipment-, or range-dependent effects, similar to what has been described for screw speed. In twin screw wet granulation, the highest possible throughput is often targeted during commercial manufacturing to reduce production time. Although increasing the throughput often only has a limited effect on granule quality, formulators should be aware that this can be associated with a higher torque, possibly shutting down the entire process. In addition, higher throughputs also require a higher drying capacity of the subsequent drying unit operation, which can be challenging when processing formulations requiring a high L/S ratio.

### 3.4. Barrel Temperature

Although the effect of barrel temperature on granule characteristics is generally limited due to a narrow evaluated range, water soluble excipients can benefit from operating at elevated barrel temperature due to the increased solubility, reducing the amount of fines [2,51,102]. Ito and Kleinebudde found that barrel temperature could be used to steer granule PSD by varying it between 30 and 90 °C. However, the exact effect also proved formulation-dependent, and these high temperatures might not be acceptable for thermosensitive compounds [82]. It should be stressed that when running at a high process torque, barrel temperature can take a long time to equilibrate. This can significantly increase the amount of blend required to reach steady state, and it is therefore advised to avoid barrel temperatures lower than 30 °C [2].

### 3.5. Screw Design

Due the fully modular setup of twin screw granulator screws and the abundance of available screw types, a broad range of screw configurations has been described in literature [52,53,103]. Consequently, the assessment of an optimized screw configuration often remains rather empirical, although the impact of screw configuration on granule characteristics and processability is widely recognized. Generally, powder is added on top of a long conveying section that transports the blend towards one or two kneading blocks, consisting of multiple kneading elements positioned at a certain offset or stagger angle (mostly 60° forward) [21,46,54,57,94,102]. Towards the end of the granulation screws, often size control/chopping/screw mixing elements are implemented to reduce the oversized fraction and obtain a more monomodal PSD [2,52,68,83]. Formulators should be aware of the large heterogeneity in available screw elements between manufacturers and hence possible screw configurations, potentially affecting method transfer between R&D and production sites.

For a more detailed overview of commonly used screw elements and the effect of screw design on granule quality attributes, the reader is referred to the reviews of Thompson [32], Bandari et al. [104], and Zhang et al. [105].

## 4. Recommendations and Research Opportunities

### 4.1. Formulation

Despite the recent developments in the field of continuous manufacturing and twin screw wet granulation, several key aspects still need to be addressed in the area of equipment and process design as well as formulation development.

The complexity of a fully integrated manufacturing line is often considered one of the major hurdles for its implementation. In traditional batch manufacturing, a large number of excipients with different functionalities is often used. When transferring such an approach to continuous manufacturing, this would translate into an excessive number of feeders, which challenges the aspect of blend uniformity. Therefore, more research is required in the field of simplified formulations. This concept was originally studied by Meier et al. for formulations containing 90% ibuprofen [22] and was later applied to a high-dosed hydrochlorothiazide formulation [106]. Recently, Vanhoorne et al. demonstrated that delta mannitol could be used as a suitable filler and binder for granulating 75% paracetamol [60]. In the future, special attention should also be dedicated towards novel (co-processed) excipients that combine several functionalities. As simplified formulations and co-processed excipients reduce the level of technical complexity, these could prove highly beneficial towards implementation of continuous manufacturing as well as facilitate drug product registration.

As previously mentioned, several excipient manufacturers are currently making efforts to develop/adapt their excipients for enhanced functionality in continuous manufacturing. Not only macromolecular properties such as bulk flow, density, and particle size should be considered in this endeavor. Although these properties are generally considered the main drivers for feeding behavior, other descriptors such as specific surface area and porosity could be influential for unit operations such as twin screw wet granulation. Apart from excipient properties, API properties should also be optimized during crystallization. As APIs regularly exhibit detrimental properties (low density, needle shape), which are currently often not optimized during drug substance development, substantial gains are still possible in this research area.

Some recent studies focused on the importance of raw material properties of excipients towards their behavior in unit operations such as feeding and twin screw granulation [46,107]. This approach should be extended towards API crystallization during drug substance manufacturing. As APIs are known as a significant risk factor to negatively impact the processability, optimizing API characteristics (particle shape, size, cohesiveness) could be of great interest, especially for continuous manufacturing lines, where the residence time in each unit operation is limited from seconds to a few minutes.

As the use of surfactants remains a largely uncovered research area, additional focus should be attributed to these excipients, focusing on the surfactant type, concentration, and addition method. Key considerations associated with the addition of surfactants should be taken into account, since these amphiphilic molecules are generally added in very low concentrations (<1%). For an average line throughput of 20 kg/h, this translates into a surfactant feed rate of less than 200 g/h, which is often not feasible with standard feeding equipment. For these purposes, the potential of micro feeders could be further evaluated. Additionally, blend homogeneity is considered critical, as these surfactants can have a significant impact on the required amount of liquid to obtain granules. Hence, it is expected that pulsations in the surfactant feeder output could propagate throughout the system if insufficient mixing is provided by the blender, resulting in pulsation of the granule quality attributes (size, strength, density, flowability). Similar challenges could also be expected when adding lubricants such as magnesium stearate before tableting.

Conventionally, water has been used as solvent in continuous twin screw wet granulation. However, given the necessary safety precautions, organic solvents could be considered to increase the applicability of this manufacturing technique. Due to the restrictions on residual solvents in drug products, class III organic solvents such as ethanol, dimethyl sulfoxide, ethyl acetate, and acetone should be preferred [108]. By selecting a proper granulation liquid, suitable for dissolving the API and/or excipients, challenging formulations with a high content of a poorly water soluble API could be processed more easily. This concept has recently been proven by Démuth et al., who manufactured low-dosed carvedilol granules by dissolving the API in ethanol [109]. In addition, the use of alternative solvents could also prevent the addition of high amounts of water or the addition of a surfactant.

### 4.2. Equipment Design and Process Control

Since GEA was the first equipment manufacturer to commercialize a full from-powder-to-tablet production line, most research papers discuss data acquired using a GEA Consigma production line. Although most manufacturing lines have similar twin screw granulator setups, significant technical differences can be observed in the design of the drying unit operation. These specific equipment designs are associated with differences in drying capacity, which can greatly influence the application range of different manufacturing lines, since drying time can be a limiting factor when applying a high L/S ratio. Therefore, comparative studies evaluating several formulations would be useful to understand to what extent equipment design impacts granule quality attributes and if these findings are formulation-dependent. As most studies to date studied twin screw wet granulation as a stand-alone unit operation, it is essential that more data become available to evaluate the interaction between the characteristics of the wet granules leaving the barrel and subsequent downstream processing (drying, milling, tableting, and coating). Therefore, it is recommended to consider holistic approaches that integrate all relevant unit operations, similar to the studies of Stauffer et al. and De Leersnyder et al. [79,110].

As previously described, most continuous lines currently still contain one or multiple mini-/semi-batch unit operations (mostly drying and tablet coating). Therefore, further research and development could be dedicated to truly continuous solutions. Apart from the continuous fluid bed dryer by L.B. Bohle mentioned above, Freund Vector also has developed a continuous drying solution by implementing a spiral dryer in its Granuformer [110,111]. Currently, a limited number of studies has been published using these truly continuous techniques, potentially limiting their industrial implementation.

In a next phase, the integration of both drug substance and drug product manufacturing should be evaluated. As continuous manufacturing generates significant process data, future research projects should also focus on management of big data and integration of artificial intelligence for process monitoring and optimization. Hence, it can be studied to what extent this can contribute to improve process understanding and a consistent high quality drug product. Implementation of soft sensors in continuous manufacturing could prove a very useful tool, as demonstrated by Rehrl et al. [112]. These data gathering and processing tools should especially be evaluated when performing long runs, a critical aspect of twin screw granulation and continuous manufacturing that has been limitedly studied so far. Additionally, the presence of dead zones and residual material (e.g., sticking to the screws or barrel wall) in each unit operation should be identified and quantified to ensure full material traceability throughout the integrated manufacturing line.

## 5. Conclusions

In the last decade, twin screw wet granulation has rapidly evolved from a promising continuous manufacturing technique to a more mature and widely adopted technique. Due to the large interest of different stakeholders such as equipment providers, global pharmaceutical companies, excipient manufacturers, and academic institutions, a transformation in the scope of research papers has taken place. Whereas initial research papers often focused on a proof of concept using a placebo formulation, more recent papers often deal with more industrially relevant topics (e.g., PAT-implementation, formulation development, control strategies, residence time distribution) where drug-loaded formulations are studied. Recent market approvals have also demonstrated the full potential of twin screw wet granulation as an alternative to conventional batch granulation techniques such as high shear granulation and fluid bed granulation. Given its well-documented advantages over batch manufacturing, further industrial adoption of twin screw wet granulation is expected in the future. Despite these recent advances, additional research is still required, particularly on aspects related to formulation development, equipment design, and process control. Comparative studies between different equipment types and mechanistic modeling could be of high interest to evaluate potentially different technical capabilities and limitations, especially when more recently introduced drying techniques are incorporated after granulation.

## Figures and Tables

**Table 1:** Overview of research papers on twin screw wet granulation evaluating formulations consisting of an API and a filler combination of MCC and lactose.

Water Soluble Filler (Grade)	Ratio Water Soluble Filler/MCC	API	Granular API Content	Reference(s)
Lactose (Pharmatose 200M)	1	Acetaminophen	5%	[12]
Lactose (Flowlac 100)	1	Acetaminophen	15%	[66]
Lactose (Pharmatose 200M)	2.33	Albendazole	50%	[67]
Lactose (Flowlac 100)	1	Caffeine	15%	[66]
Lactose (Flowlac 100)	1	Griseofulvin	15%	[66]
Lactose (Pharmatose 200M)	1	Hydrochlorothiazide	60%	[68]
Lactose (Flowlac 100)	1	Ibuprofen	15%	[66]
Lactose (Granulac 70)	1.33	Ibuprofen	30%	[69]
Lactose (Granulac 200)	1.4	Ibuprofen	51.5%	[70]
Lactose (Pharmatose 200M)	1	Mebendazole	5%	[57]
Lactose (Pharmatose 200M)	1	Mebendazole	10%	[12]
Lactose (Pharmatose 200M)	1	Mebendazole	50%	[57,59]
Lactose (Pharmatose 200M)	1	Metformin.HCl	5%	[57,71]
Lactose (Pharmatose 200M)	1	Metformin.HCl	10%	[12]
Lactose (Pharmatose 200M)	1	Metformin.HCl	50%	[57]
Lactose (Pharmatose 200M)	1	Theophylline	5%	[12]

**Table 2:** Overview of research papers on twin screw wet granulation evaluating formulations consisting of an API and a filler combination of MCC and mannitol.

Water Soluble Filler (Grade)	Ratio Water Soluble Filler/MCC	API	Granular API Content	Reference(s)
Mannitol (Pearlitol 160C)	1	Acetaminophen	25%	[29]
Mannitol (Pearlitol 160C)	1	Allopurinol	25%	[27,29]
Mannitol (Pearlitol 200SD)	1	Allopurinol	25%	[27]
Mannitol (Pearlitol 160C)	1	Metformin.HCl	25%	[29]
Mannitol (Pearlitol 160C)	2.08	Not disclosed	22%	[26]

## Data Availability

Not applicable.

## References

[1] U.S. Food and Drug Administration (2019). Quality Considerations for Continuous Manufacturing—Guidance for Industry—Draft Guidance.

[2] Portier C., Pandelaere K., Delaet U., Vigh T., Kumar A., Di Pretoro G., De Beer T., Vervaet C., Vanhoorne V. (2020). Continuous twin screw granulation: Influence of process and formulation variables on granule quality attributes of model formulations. Int. J. Pharm..

[3] Teżyk M., Milanowski B., Ernst A., Lulek J. (2016). Recent progress in continuous and semi-continuous processing of solid oral dosage forms: A review. Drug Dev. Ind. Pharm..

[4] Lee S.L., O’Connor T.F., Yang X., Cruz C.N., Chatterjee S., Madurawe R.D., Moore C.M.V., Yu L.X., Woodcock J. (2015). Modernizing Pharmaceutical Manufacturing: From Batch to Continuous Production. J. Pharm. Innov..

[5] Karttunen A.P., Hörmann T.R., De Leersnyder F., Ketolainen J., De Beer T., Hsiao W.K., Korhonen O. (2019). Measurement of residence time distributions and material tracking on three continuous manufacturing lines. Int. J. Pharm..

[6] Vercruysse J., Peeters E., Fonteyne M., Cappuyns P., Delaet U., Van Assche I., De Beer T., Remon J.P., Vervaet C. (2015). Use of a continuous twin screw granulation and drying system during formulation development and process optimization. Eur. J. Pharm. Biopharm..

[7] Madarász L., Nagy Z.K., Hoffer I., Szabó B., Csontos I., Pataki H., Démuth B., Szabó B., Csorba K., Marosi G. (2018). Real-time feedback control of twin-screw wet granulation based on image analysis. Int. J. Pharm..

[8] De Leersnyder F., Vanhoorne V., Kumar A., Vervaet C., De Beer T. (2019). Evaluation of an in-line NIR spectroscopic method for the determination of the residence time in a tablet press. Int. J. Pharm..

[9] Pauli V., Roggo Y., Kleinebudde P., Krumme M. (2019). Real-time monitoring of particle size distribution in a continuous granulation and drying process by near infrared spectroscopy. Eur. J. Pharm. Biopharm..

[10] Verstraeten M., Van Hauwermeiren D., Hellings M., Hermans E., Geens J., Vervaet C., Nopens I., De Beer T. (2018). Model-based NIR spectroscopy implementation for in-line assay monitoring during a pharmaceutical suspension manufacturing process. Int. J. Pharm..

[11] Vanhoorne V., Vervaet C. (2020). Recent progress in continuous manufacturing of oral solid dosage forms. Int. J. Pharm..

[12] Portier C., De Vriendt C., Vigh T., Di Pretoro G., De Beer T., Vervaet C., Vanhoorne V. (2020). Continuous twin screw granulation: Robustness of lactose/MCC-based formulations. Int. J. Pharm..

[13] Byrn S., Futran M., Thomas H., Jayjock E., Maron N., Meyer R.F., Myerson A.S., Thien M.P., Trout B.L. (2015). Achieving continuous manufacturing for final dosage formation: Challenges and how to meet them 20–21 May, 2014 continuous manufacturing symposium. J. Pharm. Sci..

[14] Fonteyne M., Wickström H., Peeters E., Vercruysse J., Ehlers H., Peters B.H., Remon J.P., Vervaet C., Ketolainen J., Sandler N. (2014). Influence of raw material properties upon critical quality attributes of continuously produced granules and tablets. Eur. J. Pharm. Biopharm..

[15] Liu H., Ricart B., Stanton C., Smith-Goettler B., Verdi L., O’Connor T., Lee S., Yoon S. (2019). Design space determination and process optimization in at-scale continuous twin screw wet granulation. Comput. Chem. Eng..

[16] Stauffer F., Vanhoorne V., Pilcer G., Chavez P.F., Rome S., Schubert M.A., Aerts L., De Beer T. (2018). Raw material variability of an active pharmaceutical ingredient and its relevance for processability in secondary continuous pharmaceutical manufacturing. Eur. J. Pharm. Biopharm..

[17] Kumar A., Gernaey K.V., De Beer T., Nopens I. (2013). Model-based analysis of high shear wet granulation from batch to continuous processes in pharmaceutical production—A critical review. Eur. J. Pharm. Biopharm..

[18] Pandey P., Bindra D.S., Gour S., Trinh J., Buckley D., Badawy S. (2014). Excipient–Process Interactions and their Impact on Tablet Compaction and Film Coating. J. Pharm. Sci..

[19] Van Snick B., Holman J., Vanhoorne V., Kumar A., De Beer T., Remon J.P., Vervaet C. (2017). Development of a continuous direct compression platform for low-dose drug products. Int. J. Pharm..

[20] Leane M., Pitt K., Reynolds G.K., Dawson N., Ziegler I., Szepes A., Crean A.M., Dall Agnol R. (2018). Manufacturing classification system in the real world: Factors influencing manufacturing process choices for filed commercial oral solid dosage formulations, case studies from industry and considerations for continuous processing. Pharm. Dev. Technol..

[21] Ryckaert A., Ghijs M., Portier C., Djuric D., Funke A., Vervaet C., De Beer T. (2021). The influence of equipment design and process parameters on granule breakage in a semi-continuous fluid bed dryer after continuous twin-screw wet granulation. Pharmaceutics.

[22] Meier R., Thommes M., Rasenack N., Krumme M., Moll K.P., Kleinebudde P. (2015). Simplified formulations with high drug loads for continuous twin-screw granulation. Int. J. Pharm..

[23] Shirazian S., Ismail H.Y., Singh M., Shaikh R., Croker D.M., Walker G.M. (2019). Multi-dimensional population balance modelling of pharmaceutical formulations for continuous twin-screw wet granulation: Determination of liquid distribution. Int. J. Pharm..

[24] Pishnamazi M., Casilagan S., Clancy C., Shirazian S., Iqbal J., Egan D., Edlin C., Croker D.M., Walker G.M., Collins M.N. (2019). Microcrystalline cellulose, lactose and lignin blends: Process mapping of dry granulation via roll compaction. Powder Technol..

[25] Fülöp G., Domokos A., Galata D., Szabó E., Gyürkés M., Szabó B., Farkas A., Madarász L., Démuth B., Lendér T. (2021). Integrated twin-screw wet granulation, continuous vibrational fluid drying and milling: A fully continuous powder to granule line. Int. J. Pharm..

[26] Beer P., Wilson D., Huang Z., De Matas M. (2014). Transfer from high-shear batch to continuous twin screw wet granulation: A case study in understanding the relationship between process parameters and product quality attributes. J. Pharm. Sci..

[27] Megarry A., Taylor A., Gholami A., Wikström H., Tajarobi P. (2020). Twin-screw granulation and high-shear granulation: The influence of mannitol grade on granule and tablet properties. Int. J. Pharm..

[28] Lee K.T., Ingram A., Rowson N.A. (2013). Comparison of granule properties produced using Twin Screw Extruder and High Shear Mixer: A step towards understanding the mechanism of twin screw wet granulation. Powder Technol..

[29] Kyttä K.M., Lakio S., Wikström H., Sulemanji A., Fransson M., Ketolainen J., Tajarobi P. (2020). Comparison between twin-screw and high-shear granulation—The effect of filler and active pharmaceutical ingredient on the granule and tablet properties. Powder Technol..

[30] Matsui Y., Watano S. (2018). Evaluation of Properties of Granules and Tablets Prepared by Twin-screw Continuous Granulation and Comparison of Their Properties with Those by Batch Fluidized-bed and High Shear Granulations. J. Soc. Powder Technol. Japan.

[31] Vervaet C., Remon J.P. (2005). Continuous granulation in the pharmaceutical industry. Chem. Eng. Sci..

[32] Thompson M.R. (2015). Twin screw granulation-review of current progress. Drug Dev. Ind. Pharm..

[33] Schuettgut Portal Investing in the Future: Bohle Technology Center to Be Opened. https://www.schuettgut-portal.com/newsitem/6085/investitionen-in-die-zukunft--bohle-technology-center-wird-i.html.

[34] Syntegon Xelum Continuous Manufacturing. https://www.syntegon.com/products/xelum-continuous-manufacturing.

[35] Yu L.X. Continuous Manufacturing Has a Strong Impact on Drug Quality. https://blogs.fda.gov/fdavoice/index.php/2016/04/continuous-manufacturing-has-a-strong-impact-on-drug-quality.

[36] U.S. Food and Drug Administration FDA Approves Lorlatinib for Second- or Third-Line Treatment of ALK-Positive Metastatic NSCLC. https://www.fda.gov/Drugs/InformationOnDrugs/ApprovedDrugs/ucm625027.htm.

[37] U.S. Food and Drug Administration FDA Approves Abemaciclib as Initial Therapy for HR-Positive, HER2-Negative Metastatic Breast Cancer. https://www.fda.gov/drugs/resources-information-approved-drugs/fda-approves-abemaciclib-initial-therapy-hr-positive-her2-negative-metastatic-breast-cancer.

[38] U.S. Food and Drug Administration FDA Approves New Treatment for Patients with Acute Myeloid Leukemia. https://www.fda.gov/NewsEvents/Newsroom/PressAnnouncements/ucm626443.htm.

[39] Badman C., Cooney C.L., Florence A., Konstantinov K., Krumme M., Mascia S., Nasr M., Trout B.L. (2019). Why We Need Continuous Pharmaceutical Manufacturing and How to Make It Happen. J. Pharm. Sci..

[40] U.S. Food and Drug Administration (2004). Guidance for Industry PAT—A Framework for Innovative Pharmaceutical Development, Manufacturing, and Quality Assurance.

[41] JRS Pharma (2019). Benefits of Using High-Functionality Excipients in a Continuous Manufacturing Process.

[42] Manufacturing Chemist the Rise of Independent Excipients. https://www.manufacturingchemist.com/news/article_page/The_rise_of_independent_excipients/147856.

[43] Challener C. Key Ingredients Needed to Drive the Success of Continuous Manufacturing | Pharmaceutical Technology. https://www.pharmtech.com/view/key-ingredients-needed-to-drive-the-success-of-continuous-manufacturing.

[44] DFE Pharma Continuous Manufacturing. https://www.dfepharma.com.br/Excipients/Certified-Knowledge/Knowledge-Base/ContinuousManufacturing.

[45] Vandevivere L., Portier C., Vanhoorne V., Häusler O., Simon D., De Beer T., Vervaet C. (2019). Native starch as in situ binder for continuous twin screw wet granulation. Int. J. Pharm..

[46] Vandevivere L., Denduyver P., Portier C., Häusler O., De Beer T., Vervaet C., Vanhoorne V. (2020). Influence of binder attributes on binder effectiveness in a continuous twin screw wet granulation process via wet and dry binder addition. Int. J. Pharm..

[47] Vanhoorne V., Bekaert B., Peeters E., De Beer T., Remon J.P., Vervaet C. (2016). Improved tabletability after a polymorphic transition of delta-mannitol during twin screw granulation. Int. J. Pharm..

[48] Kumar A., Alakarjula M., Vanhoorne V., Toiviainen M., De Leersnyder F., Vercruysse J., Juuti M., Ketolainen J., Vervaet C., Remon J.P. (2016). Linking granulation performance with residence time and granulation liquid distributions in twin-screw granulation: An experimental investigation. Eur. J. Pharm. Sci..

[49] Meier R., Moll K.P., Krumme M., Kleinebudde P. (2017). Impact of fill-level in twin-screw granulation on critical quality attributes of granules and tablets. Eur. J. Pharm. Biopharm..

[50] Fonteyne M., Correia A., De Plecker S., Vercruysse J., Ilić I., Zhou Q., Vervaet C., Remon J.P., Onofre F., Bulone V. (2015). Impact of microcrystalline cellulose material attributes: A case study on continuous twin screw granulation. Int. J. Pharm..

[51] Vercruysse J., Córdoba Díaz D., Peeters E., Fonteyne M., Delaet U., Van Assche I., De Beer T., Remon J.P., Vervaet C. (2012). Continuous twin screw granulation: Influence of process variables on granule and tablet quality. Eur. J. Pharm. Biopharm..

[52] Vercruysse J., Burggraeve A., Fonteyne M., Cappuyns P., Delaet U., Van Assche I., De Beer T., Remon J.P., Vervaet C. (2015). Impact of screw configuration on the particle size distribution of granules produced by twin screw granulation. Int. J. Pharm..

[53] Djuric D., Kleinebudde P. (2008). Impact of screw elements on continuous granulation with a twin-screw extruder. J. Pharm. Sci..

[54] Li H., Thompson M.R., O’Donnell K.P. (2014). Understanding wet granulation in the kneading block of twin screw extruders. Chem. Eng. Sci..

[55] Willecke N., Szepes A., Wunderlich M., Remon J.P., Vervaet C., De Beer T. (2017). Identifying overarching excipient properties towards an in-depth understanding of process and product performance for continuous twin-screw wet granulation. Int. J. Pharm..

[56] Thompson M.R., O’Donnell K.P. (2015). “Rolling” phenomenon in twin screw granulation with controlled-release excipients. Drug Dev. Ind. Pharm..

[57] Portier C., Pandelaere K., Delaet U., Vigh T., Di Pretoro G., De Beer T., Vervaet C., Vanhoorne V. (2020). Continuous twin screw granulation: A complex interplay between formulation properties, process settings and screw design. Int. J. Pharm..

[58] Vandevivere L., Vangampelaere M., Portier C., de Backere C., Häusler O., De Beer T., Vervaet C., Vanhoorne V. (2021). Identifying critical binder attributes to facilitate binder selection for efficient formulation development in a continuous twin screw wet granulation process. Pharmaceutics.

[59] Portier C., Vigh T., Di Pretoro G., De Beer T., Vervaet C., Vanhoorne V. (2020). Continuous twin screw granulation: Impact of binder addition method and surfactants on granulation of a high-dosed, poorly soluble API. Int. J. Pharm..

[60] Vanhoorne V., Almey R., De Beer T., Vervaet C. (2020). Delta-mannitol to enable continuous twin-screw granulation of a highly dosed, poorly compactable formulation. Int. J. Pharm..

[61] Willecke N., Szepes A., Wunderlich M., Remon J.P., Vervaet C., De Beer T. (2018). A novel approach to support formulation design on twin screw wet granulation technology: Understanding the impact of overarching excipient properties on drug product quality attributes. Int. J. Pharm..

[62] Harting J., Kleinebudde P. (2018). Development of an in-line Raman spectroscopic method for continuous API quantification during twin-screw wet granulation. Eur. J. Pharm. Biopharm..

[63] Rowe R.C., Sheskey P.J., Quinn M.E. (2009). Handbook of Pharmaceutical Excipients.

[64] Thoorens G., Krier F., Leclercq B., Carlin B., Evrard B. (2014). Microcrystalline cellulose, a direct compression binder in a quality by design environment—A review. Int. J. Pharm..

[65] Ridgway C., Bawuah P., Markl D., Zeitler J.A., Ketolainen J., Peiponen K.E., Gane P. (2017). On the role of API in determining porosity, pore structure and bulk modulus of the skeletal material in pharmaceutical tablets formed with MCC as sole excipient. Int. J. Pharm..

[66] Li H., Thompson M.R., O’Donnell K.P. (2015). Examining drug hydrophobicity in continuous wet granulation within a twin screw extruder. Int. J. Pharm..

[67] Kashani Rahimi S., Paul S., Sun C.C., Zhang F. (2020). The role of the screw profile on granular structure and mixing efficiency of a high-dose hydrophobic drug formulation during twin screw wet granulation. Int. J. Pharm..

[68] Verstraeten M., Van Hauwermeiren D., Lee K., Turnbull N., Wilsdon D., am Ende M., Doshi P., Vervaet C., Brouckaert D., Mortier S.T.F.C. (2017). In-depth experimental analysis of pharmaceutical twin-screw wet granulation in view of detailed process understanding. Int. J. Pharm..

[69] Arndt O.R., Baggio R., Adam A.K., Harting J., Franceschinis E., Kleinebudde P. (2018). Impact of Different Dry and Wet Granulation Techniques on Granule and Tablet Properties: A Comparative Study. J. Pharm. Sci..

[70] Miyazaki Y., Lenhart V., Kleinebudde P. (2020). Switch of tablet manufacturing from high shear granulation to twin-screw granulation using quality by design approach. Int. J. Pharm..

[71] Portier C., Vigh T., Di Pretoro G., Leys J., Klingeleers D., De Beer T., Vervaet C., Vanhoorne V. (2021). Continuous twin screw granulation: Impact of microcrystalline cellulose batch-to-batch variability during granulation and drying—A QbD approach. Int. J. Pharm. X.

[72] Harting J., Kleinebudde P. (2019). Optimisation of an in-line Raman spectroscopic method for continuous API quantification during twin-screw wet granulation and its application for process characterisation. Eur. J. Pharm. Biopharm..

[73] Vanhoorne V., Janssens L., Vercruysse J., De Beer T., Remon J.P., Vervaet C. (2016). Continuous twin screw granulation of controlled release formulations with various HPMC grades. Int. J. Pharm..

[74] Schmidt A., de Waard H., Moll K.P., Kleinebudde P., Krumme M. (2018). Simplified end-to-end continuous manufacturing by feeding API suspensions in twin-screw wet granulation. Eur. J. Pharm. Biopharm..

[75] El Hagrasy A.S., Hennenkamp J.R., Burke M.D., Cartwright J.J., Litster J.D. (2013). Twin screw wet granulation: Influence of formulation parameters on granule properties and growth behavior. Powder Technol..

[76] Hwang K.-M., Cho C.-H., Yoo S.-D., Cha K.-I., Park E.-S. (2019). Continuous twin screw granulation: Impact of the starting material properties and various process parameters. Powder Technol..

[77] Paul S., Chang S.Y., Dun J., Sun W.J., Wang K., Tajarobi P., Boissier C., Sun C.C. (2018). Comparative analyses of flow and compaction properties of diverse mannitol and lactose grades. Int. J. Pharm..

[78] Stauffer F., Vanhoorne V., Pilcer G., Chavez P.F., Vervaet C., De Beer T. (2019). Managing API raw material variability during continuous twin-screw wet granulation. Int. J. Pharm..

[79] Stauffer F., Vanhoorne V., Pilcer G., Chavez P.F., Vervaet C., De Beer T. (2019). Managing API raw material variability in a continuous manufacturing line—Prediction of process robustness. Int. J. Pharm..

[80] Ritala M., Jungersen O., Holm P., Schæfer T., Kristensen H.G. (1986). A comparison between binders in the wet phase of granulation in a high shear mixer. Drug Dev. Ind. Pharm..

[81] Wu Y., Levons J., Narang A.S., Raghavan K., Rao V.M. (2011). Reactive impurities in excipients: Profiling, identification and mitigation of drug-excipient incompatibility. AAPS PharmSciTech.

[82] Ito A., Kleinebudde P. (2019). Influence of granulation temperature on particle size distribution of granules in twin-screw granulation (TSG). Pharm. Dev. Technol..

[83] Vanhoorne V., Vanbillemont B., Vercruysse J., De Leersnyder F., Gomes P., De Beer T., Remon J.P., Vervaet C. (2016). Development of a controlled release formulation by continuous twin screw granulation: Influence of process and formulation parameters. Int. J. Pharm..

[84] Roggo Y., Pauli V., Jelsch M., Pellegatti L., Elbaz F., Ensslin S., Kleinebudde P., Krumme M. (2020). Continuous manufacturing process monitoring of pharmaceutical solid dosage form: A case study. J. Pharm. Biomed. Anal..

[85] Dhenge R.M., Cartwright J.J., Hounslow M.J., Salman A.D. (2012). Twin screw wet granulation: Effects of properties of granulation liquid. Powder Technol..

[86] Cao Q.R., Choi Y.W., Cui J.H., Lee B.J. (2005). Formulation, release characteristics and bioavailability of novel monolithic hydroxypropylmethylcellulose matrix tablets containing acetaminophen. J. Control. Release.

[87] Malmsten M. (2002). Surfactants and Polymers in Drug Delivery.

[88] Tadros T.F. (2005). Applied Surfactants: Principles and Applications.

[89] Thompson M.R., Weatherley S., Pukadyil R.N., Sheskey P.J. (2012). Foam granulation: New developments in pharmaceutical solid oral dosage forms using twin screw extrusion machinery. Drug Dev. Ind. Pharm..

[90] Thompson M.R., Mu B., Sheskey P.J. (2012). Aspects of foam stability influencing foam granulation in a twin screw extruder. Powder Technol..

[91] Rocca K.E., Weatherley S., Sheskey P.J., Thompson M.R. (2015). Influence of filler selection on twin screw foam granulation. Drug Dev. Ind. Pharm..

[92] Li H., Thompson M.R., O’Donnell K.P. (2015). Progression of wet granulation in a twin screw extruder comparing two binder delivery methods. AIChE J..

[93] Weatherley S., Thompson M.R., Sheskey P.J. (2013). A study of foam granulation and wet granulation in a twin screw extruder. Can. J. Chem. Eng..

[94] Seem T.C., Rowson N.A., Ingram A., Huang Z., Yu S., de Matas M., Gabbott I., Reynolds G.K. (2015). Twin screw granulation—A literature review. Powder Technol..

[95] Keleb E.I., Vermeire A., Vervaet C., Remon J.P. (2004). Twin screw granulation as a simple and efficient tool for continuous wet granulation. Int. J. Pharm..

[96] Schmidt A., de Waard H., Moll K.-P., Krumme M., Kleinebudde P. (2016). Quantitative Assessment of Mass Flow Boundaries in Continuous Twin-screw Granulation. Chimia.

[97] Dhenge R.M., Fyles R.S., Cartwright J.J., Doughty D.G., Hounslow M.J., Salman A.D. (2010). Twin screw wet granulation: Granule properties. Chem. Eng. J..

[98] Djuric D., Van Melkebeke B., Kleinebudde P., Remon J.P., Vervaet C. (2009). Comparison of two twin-screw extruders for continuous granulation. Eur. J. Pharm. Biopharm..

[99] Thompson M.R., Sun J. (2010). Wet granulation in a twin-screw extruder: Implications of screw design. J. Pharm. Sci..

[100] Dhenge R.M., Cartwright J.J., Doughty D.G., Hounslow M.J., Salman A.D. (2011). Twin screw wet granulation: Effect of powder feed rate. Adv. Powder Technol..

[101] Djuric D., Kleinebudde P. (2010). Continuous granulation with a twin-screw extruder: Impact of material throughput. Pharm. Dev. Technol..

[102] Fonteyne M., Vercruysse J., Díaz D.C., Gildemyn D., Vervaet C., Remon J.P., Beer T. (2013). De Real-time assessment of critical quality attributes of a continuous granulation process. Pharm. Dev. Technol..

[103] Li J., Pradhan S.U., Wassgren C.R. (2019). Granule transformation in a twin screw granulator: Effects of conveying, kneading, and distributive mixing elements. Powder Technol..

[104] Bandari S., Nyavanandi D., Kallakunta V.R., Janga K.Y., Sarabu S., Butreddy A., Repka M.A. (2020). Continuous twin screw granulation—An advanced alternative granulation technology for use in the pharmaceutical industry. Int. J. Pharm..

[105] Zhang Y., Liu T., Kashani-Rahimi S., Zhang F. (2021). A review of twin screw wet granulation mechanisms in relation to granule attributes. Drug Dev. Ind. Pharm..

[106] Meier R., Moll K.-P., Krumme M., Kleinebudde P. (2017). Simplified, High Drug-Loaded Formulations Containing Hydrochlorothiazide for Twin-Screw Granulation. Chemie Ing. Tech..

[107] Van Snick B., Kumar A., Verstraeten M., Pandelaere K., Dhondt J., Di Pretoro G., De Beer T., Vervaet C., Vanhoorne V. (2019). Impact of material properties and process variables on the residence time distribution in twin screw feeding equipment. Int. J. Pharm..

[108] European Directorate for the Quality of Medicine (2018). European Pharmacopoeia 10.0: 5.4 Residual Solvents.

[109] Démuth B., Fülöp G., Kovács M., Madarász L., Ficzere M., Köte Á., Szabó B., Nagy B., Balogh A., Csorba K. (2020). Continuous manufacturing of homogeneous ultralow-dose granules by twin-screw wet granulation. Period. Polytech. Chem. Eng..

[110] De Leersnyder F., Vanhoorne V., Bekaert H., Vercruysse J., Ghijs M., Bostijn N., Verstraeten M., Cappuyns P., Van Assche I., Vander Heyden Y. (2018). Breakage and drying behaviour of granules in a continuous fluid bed dryer: Influence of process parameters and wet granule transfer. Eur. J. Pharm. Sci..

[111] Dahlgren G., Tajarobi P., Simone E., Ricart B., Melnick J., Puri V., Stanton C., Bajwa G. (2019). Continuous Twin Screw Wet Granulation and Drying—Control Strategy for Drug Product Manufacturing. J. Pharm. Sci..

[112] Rehrl J., Karttunen A.P., Nicolaï N., Hörmann T., Horn M., Korhonen O., Nopens I., De Beer T., Khinast J.G. (2018). Control of three different continuous pharmaceutical manufacturing processes: Use of soft sensors. Int. J. Pharm..

